# The hidden burden of lysosomal dysfunction: visual decline and microphthalmia in Hunter syndrome

**DOI:** 10.1055/s-0045-1809405

**Published:** 2025-06-21

**Authors:** Mateen Sheikh, Ibrahim Thein, Kevin J. Abrams, Leonardo Furtado Freitas

**Affiliations:** 1 Florida International University, Herbert Wertheim College of Medicine, Department of Medicine, Miami FL, United States.; 2 Baptist Health South Florida, Division of Clinical Neuroradiology, Department of Radiology, Miami FL, United States.; 3 Florida International University, Herbert Wertheim College of Medicine, Clinical, Miami FL, United States.

A 43-year-old male patient with Hunter syndrome (also known as *mucopolysaccharidosis type II* , MPS II) presented with progressive visual impairment and bilateral upper limb weakness. Multimodal imaging revealed posterior scleral thickening, optic disc edema, and photoreceptor loss, indicating a progressive ophthalmopathy associated with lysosomal storage dysfunction.

**Figure 1:**
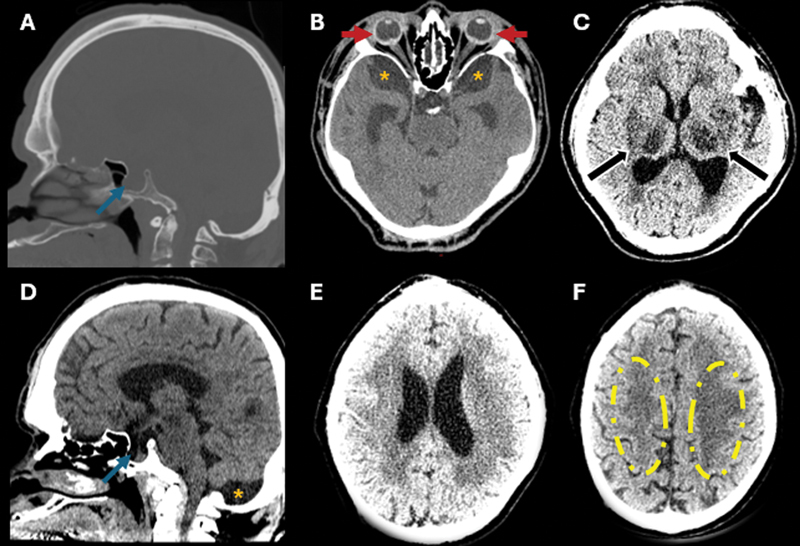
Computed tomography (CT) scan of the head in bone window ( **A** ) and soft tissue window ( **B–F** ). Intracranial stigmata of mucopolysaccharidosis with enlarged and partially-empty sella turcica (blue arrows), circumferential thickening of the bilateral sclera and microphthalmia (red arrows), prominent cerebrospinal fluid (CSF) in the middle cranial fossae and mega cisterna magna (orange asterisks), ventriculomegaly, and diffuse white matter abnormalities (yellow dashed circles) with multiple prominent perivascular spaces (PVSs; black arrows), predominantly in the posterior subinsular region, putamina, and thalami. Additionally, there is mild diffuse brain atrophy.

While glycosaminoglycan accumulation in the ocular structures has been rarely reported, 1 2 3 the present is the first report of its progression to chronic ophthalmopathy with microphthalmia. Neuroimaging ( Figures 1 2 3 ) demonstrated cervical spinal stenosis with potential dynamic myelopathy, likely contributing to limb weakness. 4 5

**Figure 2:**
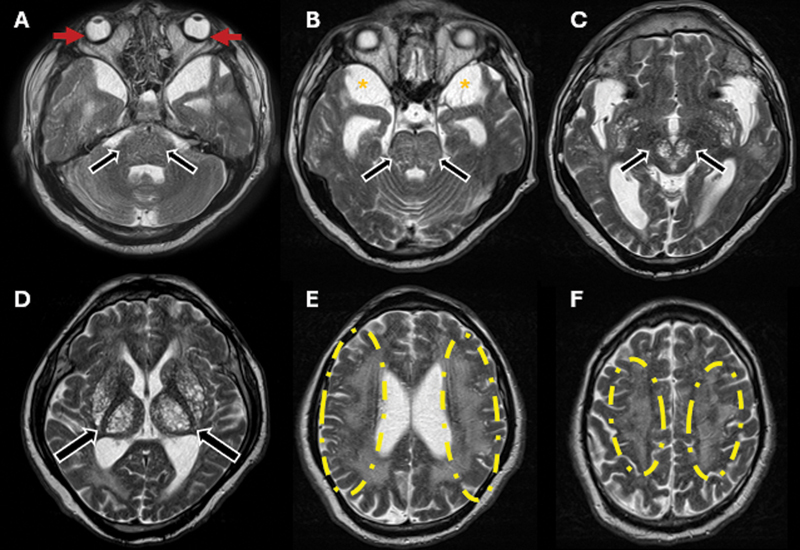
Brain magnetic resonance imaging (MRI) scan, axial T2-weighted images ( **A–F** ). Increased conspicuity of intracranial stigmata of mucopolysaccharidosis. Notable findings include circumferential scleral thickening and microphthalmia (red arrows), attributed to hypointense material representing glycosaminoglycan accumulation. There was also prominent CSF spaces in the middle cranial fossae (orange asterisks), ventriculomegaly, and diffuse white matter abnormalities (yellow dashed circles) with multiple PVSs (black arrows), predominantly in the posterior subinsular region, putamina, and thalami, extending into the upper midbrain. This microcystic appearance is known as the *honeycomb* pattern.

**Figure 3:**
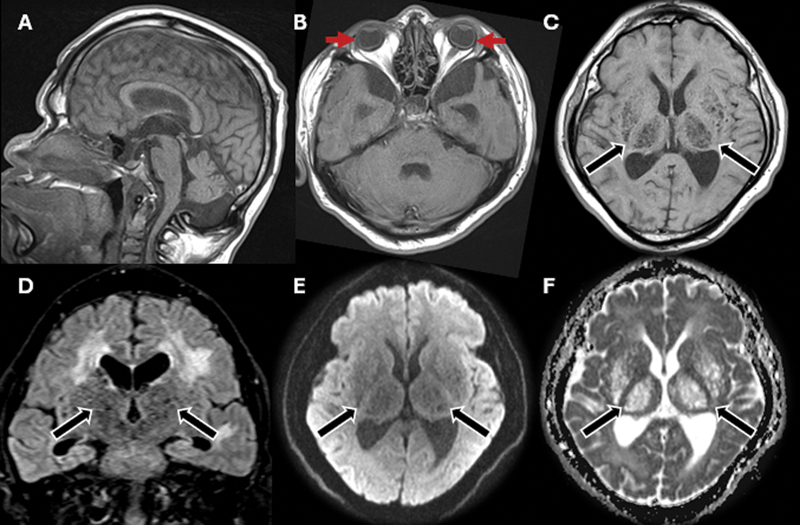
Brain MRI scan, including sagittal T1-weighted ( **A** ), axial T1-weighted ( **B–C** ), coronal fluid-attenuated inversion recovery (FLAIR) ( **D** ), axial diffusion-weighted imaging ( **E** ), and apparent diffusion coefficient (ADC) map ( **F** ). Redemonstration of circumferential scleral thickening and microphthalmia (red arrows). Multiple PVSs (black arrows) with a honeycomb pattern exhibiting signal suppression on T1- and FLAIR-weighted images, along with facilitated diffusion. White matter abnormalities show FLAIR hyperintensity, suggesting gliosis and/or demyelination secondary to lysosomal storage dysfunction and neuronal impairment.

The case herein reported highlights the need for early ophthalmologic and neuroradiologic surveillance to prevent irreversible visual and neurological deterioration in Hunter syndrome.
